# Correction: Beyond Two-Stage Models for Lung Carcinogenesis in the Mayak Workers: Implications for Plutonium Risk

**DOI:** 10.1371/journal.pone.0141748

**Published:** 2015-10-23

**Authors:** The PLOS ONE Staff

## Notice of Republication

This article was republished on October 14, 2015, to correct formatting errors in the figures and tables. The publisher apologizes for the errors. Please download this article again to view the correct version. The originally published, uncorrected article and the republished, corrected article are provided here for reference.

## Supporting Information

S1 File Originally published, uncorrected article. (PDF)

S2 File Republished, corrected article. (PDF)
