# Supplementary material for: Correction: Beyond Two-Stage Models for Lung Carcinogenesis in the Mayak Workers: Implications for Plutonium Risk
Source: PLoS One. 2015 Oct 23;10(10):e0141748. doi: 10.1371/journal.pone.0141748 (PMC4619685; doi:10.1371/journal.pone.0141748)
Supplement: S2 File — (PDF) [file pone.0141748.s002.pdf]

RESEARCH ARTICLE

Beyond Two-Stage Models for Lung Carcinogenesis in the Mayak Workers: Implications for Plutonium Risk

Sascha Zöllner[1]*, Mikhail E. Sokolnikov[2], Markus Eidemüller[1]

1 Helmholtz Zentrum München, Institute of Radiation Protection, Neuherberg (Germany), **2** Southern Urals Biophysics Institute, Ozyorsk (Russia)

* sascha.zoellner@helmholtz-muenchen.de

CrossMark
click for updates

OPEN ACCESS

Citation: Zöllner S, Sokolnikov ME, Eidemüller M (2015) Beyond Two-Stage Models for Lung Carcinogenesis in the Mayak Workers: Implications for Plutonium Risk. PLoS ONE 10(5): e0126238. doi:10.1371/journal.pone.0126238

Academic Editor: Olga Y Gorlova, Geisel School of Medicine at Dartmouth College, UNITED STATES

Received: December 16, 2014

Accepted: March 31, 2015

Published: May 22, 2015

Copyright: © 2015 Zöllner et al. This is an open access article distributed under the terms of the Creative Commons Attribution License, which permits unrestricted use, distribution, and reproduction in any medium, provided the original author and source are credited.

Data Availability Statement: The contact address for the data is: Ozyorsk, Russian Federation, 456780, Ozyorskoye Shosse 19. sokolnikov@subi.su

Funding: This work was supported by the European Commission under FP7 project no. 269553 (EpiRadBio, http://cordis.europa.eu/fp7/home_en.html). The funders had no role in study design, data collection and analysis, decision to publish, or preparation of the manuscript.

Competing Interests: The authors have declared that no competing interests exist.

Abstract

Mechanistic multi-stage models are used to analyze lung-cancer mortality after Plutonium exposure in the Mayak-workers cohort, with follow-up until 2008. Besides the established two-stage model with clonal expansion, models with three mutation stages as well as a model with two distinct pathways to cancer are studied. The results suggest that three-stage models offer an improved description of the data. The best-fitting models point to a mechanism where radiation increases the rate of clonal expansion. This is interpreted in terms of changes in cell-cycle control mediated by bystander signaling or repopulation following cell killing. No statistical evidence for a two-pathway model is found. To elucidate the implications of the different models for radiation risk, several exposure scenarios are studied. Models with a radiation effect at an early stage show a delayed response and a pronounced drop-off with older ages at exposure. Moreover, the dose-response relationship is strongly nonlinear for all three-stage models, revealing a marked increase above a critical dose.

Introduction

Cancer is a genetic disease. In the widely held theory of somatic evolution [1], a cell's path toward the malignant state is portrayed as a series of mutations or epigenetic events, lending it successive selective advantages. These advantages, as summarized in the "hallmarks of cancer" [2], essentially amount to an increasingly uncontrolled proliferation.

Those essential features—mutations accompanied by proliferation—have long been identified as key ingredients in modeling carcinogenesis. Beginning with the seminal multi-step models by Armitage/Doll and Nordling [3, 4], this eventually led to the stochastic two-stage model with clonal expansion due to Moolgavkar, Venzon, and Knudson [5, 6], which has by now become an established tool to understand and predict cancer risk [7–9].

What fundamentally distinguishes such mechanistic models from conventional epidemiological ones is that they do not directly model the endpoint—say, the cancer mortality rate—but rather the process thought to lead to it, parametrized via mutation and proliferation rates.

This may prove useful especially when the mortality rate is highly convoluted by an exposure to carcinogens such as ionizing radiation, as is the case in this study.

Such a mechanistic approach can be readily generalized so as to build in known biological effects, such as multiple genetic pathways [7, 10] or a more realistic number of stages [11, 12]. Indeed, for colorectal cancer, where the understanding of the cellular mechanisms is comparatively advanced [13, 14], a number of extended models have been put forward to account for the role of genomic instability [10, 15–17], the rather large number of premalignant stages [12, 18], as well as the intricate dynamics during progression [19].

By contrast, far less is known regarding other cancer types. For lung cancer, mechanistic modeling studies are abundant but have focused almost exclusively on the two-stage model. These indicate that for the two main risk factors, smoking [20–22] and α-particle radiation (most relevant being Radon decay products) [23–32], the best description is afforded by an enhancement of the proliferation rate of premalignant cells. However, for radiation, this conclusion has been disputed [33] because it lacks a conclusive biological mechanism, in contrast to the accepted mutagenic effect of radiation. This debate has been further sparked by the single analysis to date going beyond the two-stage model [34]. Comparing fits to the Colorado-miners data using the two-stage model with those from a subclass of three-stage models, the authors suggested that a proliferation effect were confined to the two-stage model, whereas a better fit quality was achieved within a three-stage framework with a mutational radiation action.

The objective of this paper is to perform a comparative analysis of lung-cancer risk associated with α-radiation using different multi-stage models—specifically two- and three-stage models as well as a two-pathway model for (radiation-induced) genomic instability. Our goal is to identify the mechanisms of radiation action suggested by those models, as well as to lay out to what extent their predicted radiation risks are consistent. To this end, we apply these models to the Mayak-workers cohort [35, 36]. These workers, employed at the formerly Soviet Plutonium-production plant, have been exposed to substantial doses of ^{239}Pu via inhalation and exhibit a large number of lung-cancer deaths, 895 in total [37]. A notable feature of Plutonium exposure is its strong protraction, which might facilitate the assessment of risk on the long time scales relevant to indoor Radon. Furthermore, information on the strongest risk factor, smoking, is available for most workers. We will show that certain three-stage models give an improved description of the data, and we elucidate how these lead to predictions for the risk that are qualitatively different from both two-stage mechanistic and standard descriptive models.

Materials and Methods

Mayak-workers cohort

The Mayak-workers cohort comprises nuclear workers at the Mayak Plutonium-production facility at Ozyorsk, Russia [35]. The current follow-up includes all years 1948–2008 and comprises 25,757 members, cf. Ref. [37] for a comprehensive overview.

Many of the workers have been exposed to Plutonium-239, predominantly through inhalation. These internal doses have been assessed via urine measurements combined with biokinetic modeling for about 40% of workers in the plants at risk [38]. Exposure to external gamma radiation has been recorded via film-badge dosimeters [39], and average annual dose rates are available for all workers. Furthermore, for most workers, information exists on smoking status (non-/ever-smoker) as well as on alcohol consumption (teetotaler/light/medium/heavy/chronic), see below. Owing to pronounced smoking and Plutonium-inhalation patterns, the

dominant cancer-mortality endpoint is lung cancer (defined here as ICD-9 code 162), with a total of 895 mortality cases.

Cohort definition. To obtain a sufficiently homogeneous data set amenable to mechanistic modeling, several selection criteria have been applied, similar to previous studies [31, 32]. Our reduced (sub)cohort excludes females, since these make up less than 25% of the whole cohort and exhibit very different mortality rates. Moreover, full information is required on smoking/alcohol status and annual internal dose(rate)s—i.e., ^{239}Pu doses must be measured or assumed to vanish (for workers outside the radiochemical and Pu plants). Although missing risk-factor information may be taken into account via additional fit categories, we found that this does not reduce the parameter uncertainties, presumably due to the noise introduced this way.

Finally, the follow-up period is restricted as follows. If a Pu measurement has been performed at time t_{Pu}, then the entry date is set to two years after the measurement date, t_{Pu} + 2a. This is done to avoid selection bias, specifically healthy-survivor effects (due to extended follow-up periods for persons surviving until t_{Pu}) and diagnostic bias (in case the measurement has been caused by imminent health problems). To ensure complete follow-up, the exit date is cut off at the end of 2008 or, in the case of migrants, 2003.

The reduced cohort includes 8,604 persons and 388 lung-cancer deaths.

Risk factors. Let us briefly highlight some aspects of the major risk factors (see Ref. [37] for details). The main interest here is in internal radiation, with measured nonzero doses available for 3,667 persons. Due to slow degradation in the lungs and the long half-life of ^{239}Pu, exposures are highly protracted: First exposure peaks around age 20, and typically continues until the end of follow-up. Cumulative lung doses are well described by a log-normal distribution. Among those measured, it is peaked at 4mGy, much below the mean dose $\bar{D} = 0.12\mathrm{Gy}$. Restricted to lung-cancer cases, the dose distribution is shifted to higher values, with $\bar{D} = 0.44\mathrm{Gy}$ and lower/upper 5% quantiles of 7.6mGy and 2.3Gy.

The overall smoking fraction is about 3/4. Alcohol status, although not known to be a risk factor for lung cancer, may serve as an indicator for smoking habits because the fraction of smokers increases with alcohol consumption. We group heavy/chronic drinkers as one category, $a = 1$, and otherwise set $a = 0$.

As with any cancer, age is a crucial intrinsic risk factor. The ages of cohort members range broadly between about 18 and 81 years, as defined by the lower/upper 5% quantiles of entry/exit age, with an average of 27 years spent in the cohort. By contrast, 90% of lung-cancer cases are found only between ages 49–78, with a mean cancer age of 65 years.

Ethics statement. The study of the Mayak-workers cohort has been reviewed and approved by the Southern Urals Biophysics Institute's Review Board for issues related to privacy and personal data protection. All patient records were anonymized and de-identified prior to analysis.

Statistical analysis

Different classes of multi-stage models with clonal expansion are applied to the Mayak data. Specifically, these are two- and three-stage models as well as a model with two distinct pathways. In the following, we will sketch the basic assumptions underlying these mechanistic models and some properties of their solutions. We then discuss how external risk factors, such as radiation, are included in this framework, before laying out the procedure for model fitting and selection.

Multi-stage models. The common rationale of all mechanistic models studied here is to radically reduce the complexity of carcinogenesis to essentially two key processes: (i) *mutations*

—or, generally, a series of (epi-)genetic transitions from healthy via pre-malignant to malignant stem-like cells—and (ii) *proliferation*(i.e., symmetric division; cell inactivation or death) of pre-malignant cells with a selective advantage [40]. Note that this simplified single-cell picture does not explicitly include cellular interactions. In particular, non-stem cells are not taken into account.

Mathematically, this is modeled as continuous-time Markov processes for the (stochastic) numbers of cells, $X_i(t)$, at the different stages ($i = 0, \ldots, k$). Specifically, at age $t = 0$, one starts with $X_0 \equiv N$ healthy cells, which can make a transition (modeled as a Poisson process) with rate μ_0 to a first, premalignant stage, X_1. These cells can then undergo a birth/death process with rates α_1/β_1, leading to a net proliferation rate $\gamma_1 \approx \alpha_1 - \beta_1$. Further transitions eventually lead to malignant cells, X_k. The occurrence of the first malignant cell is assumed to lead to cancer after an effective lag time t_{lag}, typically on the order of a few years. A cartoon depiction is shown in Fig 1; for $k = 2$, this corresponds to the standard two-stage model with clonal expansion (TSCE).

The mathematical model above can be solved for the survival function $S(t)$ and, equivalently, the hazard (here: lung-cancer mortality rate), $h = -\frac{d}{dt} lnS$, using the method of characteristics [41]. For the two-stage model, assuming rate parameters to be piecewise age-independent, an exact closed-form solution can be attained [42]. In the general case, an extension of this solution is valid approximately if age bins are small enough for intermediate-cell numbers to change slowly.

As an illustration, let us highlight some generic features shared by all such multistage models. At earlier ages, the hazard is well described by a deterministic model, $h \simeq \mu_{k-1}\bar{X}_{k-1}$, in terms of the mean numbers of cells, $\dot{\bar{X}}_j = \mu_{j-1}\bar{X}_{j-1} + \gamma_j\bar{X}_j$ [41]. This leads to an initially polynomial growth, $h(t) \simeq + N\mu_0 \cdots \mu_{k-1} t^{k-1}/(k-1)!$, followed by a rapid proliferation-driven phase, $h(t) = \mathcal{O}(e^{\gamma t})$, where γ denotes the maximum growth rate. However, this deterministic approximation fails to account for the effective reduction in available premalignant cells as new malignancies arise: Whenever a person reaches the cancer endpoint, those cells can no longer lead to further cancer cases. At older ages, approximately around the mean cancer age, a steady state is reached between growth and effective "loss" of premalignant cells [41], and the hazard levels off to a constant limit, $h_\infty \sim N\mu_0\, \gamma_1/\alpha_1$.

These borderline cases also illustrate a more general point: Not all biological rates can be determined from fits to the cancer data alone. Generically, the hazard only depends on certain parameter combinations [43]. The combinations chosen for the fits here are shown in Tables 3 and 4. For example, in the two-stage case, $N\mu_0\, \mu_1$ may be interpreted as an overall scale factor for the hazard; $\gamma \equiv \alpha_1 - \beta_1 - \mu_1$ is essentially the net proliferation rate and yields the system's (inverse) time scale, whereas $\alpha_1\, \mu_1$ effectively reduces the mean cancer age, at which saturation of the hazard sets in.

In the discussion so far, we have tacitly assumed a linear series of transitions for simplicity. However, we also test a model where mutations may occur along two different pathways, as sketched in Fig 1(bottom). Such a model has been introduced by Little *et al.* [10] to account for genomic instability, inspired by models for colon cancer [14]. The underlying idea is that a second path, activated via transition rates σ_j, corresponds to the loss of a gene involved in maintaining genomic integrity. This may lead to mutation rates much larger than for the genomically stable (upper) path, $\mu_j^{GI} \gg \mu_j$ [44]. Despite the more complex topology, the model equations are constructed and solved using exactly the same principles as outlined above.

Risk modeling. In the framework of multi-stage models, the hazard is fully determined by the (generally time-dependent) mutation and growth rates. These parameters are essentially assumed to have an age-independent background value which may be modified by external risk

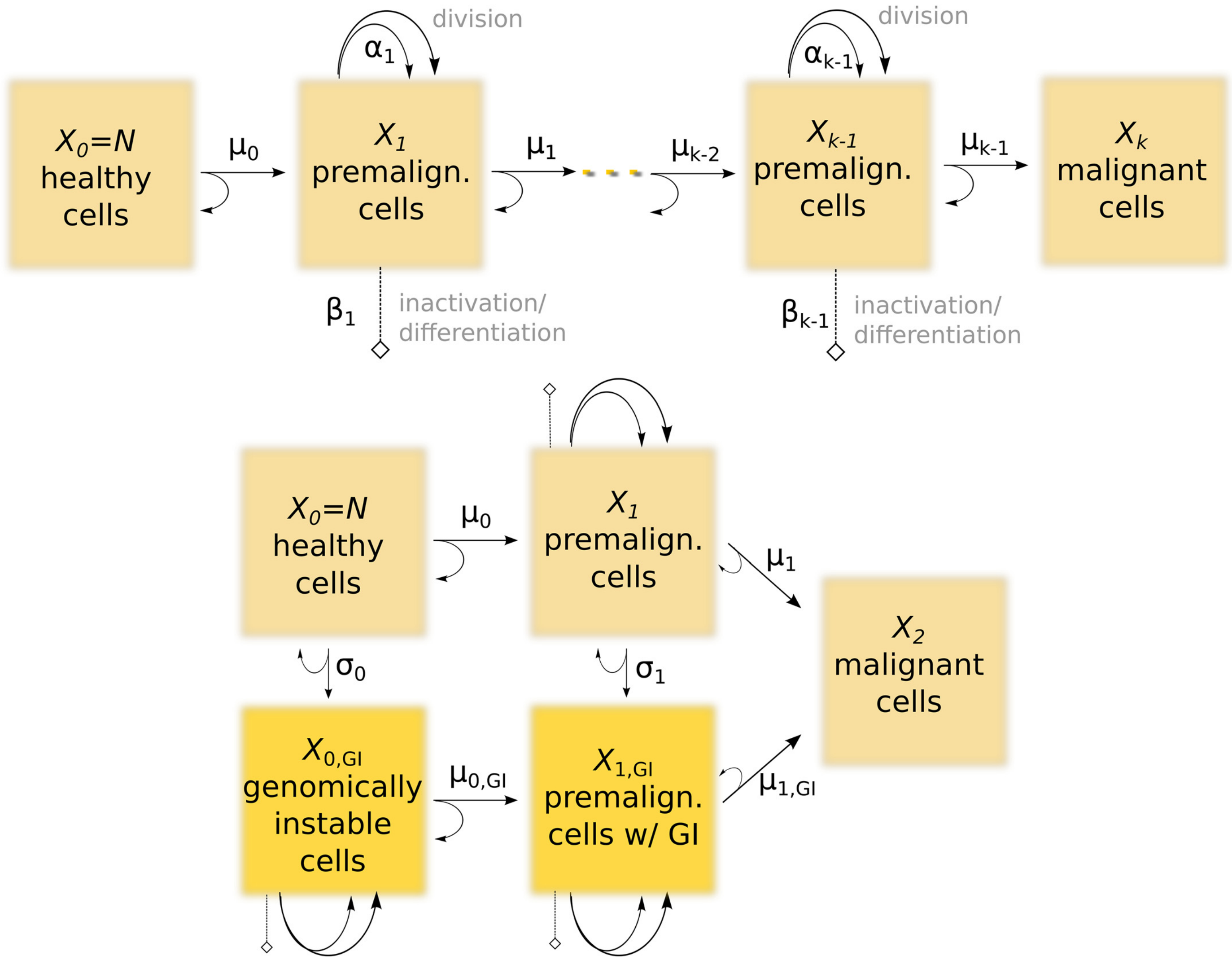

Fig 1. Schematic structure of a *k*-stage model (top) and the two-path model studied in this paper (below). Here, X_j denotes the stochastic number of cells at stage j, with arrows indicating transitions at rates μ_j, etc. (see text). Cancer is assumed to occur once the first malignant cell appears, with latency period $t_{lag} \sim 5$ years.

doi:10.1371/journal.pone.0126238.g001

factors such as radiation dose rate, $d(t)$, and smoking, $s(t)$, here assumed to start from 18 years on. In practice, both risk risk factors are allowed to independently increase any of the rate parameters $\vartheta_l \in \{\mu_0, \mu_1, \ldots, \gamma_1, \ldots\}$ in a suitable parametrization, e.g. $\vartheta_l(d)$; from all possible combinations $\{\vartheta_l(d), \vartheta_{l'}(s)\}$, the best-fitting model is selected. Depending on which of these rates show a radiation effect, the radiation risk varies in a characteristic fashion with age [42], as well as with modifiers such as duration of, or age at, exposure.

This is in marked contrast to conventional descriptive models employed in radiation epidemiology [45], which we also use to benchmark our results. In a descriptive model, the hazard function is modeled directly—rather than its underlying mechanism. Here we use the

Table 1. Synopsis of the best models in this study, along with figures of merit for their goodness of fit (see text for details). The columns labeled *d* and *s* indicate the model's parametric dependence on dose (rate) and smoking-related confounders; e.g., $\gamma = \gamma^{(0)}(s) + \delta\gamma(d)$ for the TSCE model.

Model	*s*	*d*	# Parameters[a]	Deviance	ΔAIC
descriptive	ψ_{rf}	ERR(*D*)	9	4770.3	0
TSCE	γ	γ	10	4760.7	–7.6
3SCE(A)	μ_1	γ_1	11	4749.9	–16.4
3SCE(B)	γ_2	γ_1	11	4756.7	–9.6
3SCE(C)	γ_1	γ_2	11	4757.7	–8.6

[a] Counting parameters for background, smoking/alcohol, birthyear, and Pu; see Appendix.

doi:10.1371/journal.pone.0126238.t001

conventional parametrization [37]

$$h(t) = h_{\rm bsl}(t)[1 + {\rm ERR}(D, t, \ldots)], \quad (1)$$

where the baseline, $h_{\rm bsl}(t) = e^{\psi(t)+\psi_{\rm rf}}$, is factored into terms for age-dependent background, $\psi(t) \equiv \sum_{j=0}^{2} c_j \ln^j(\frac{t}{60{\rm a}})$, and other risk factors, $\psi_{\rm rf}$ (birth year, smoking and alcohol, etc.). The excess relative risk, ERR, is factored into dose-response shape—typically a function of cumulative dose, $D(t - t_{\rm lag})$—and time-dependent modifiers such as attained age or age at exposure; see Appendix. Generally, to test specific covariables, we first use categorical fits so as to explore the qualitative dependence on this covariable, before constructing adequate analytic parametrizations.

Fit procedure. All model parameters ϑ are estimated by maximizing their likelihood, $L(\vartheta) = \prod_i \ell_i(\vartheta)$, constructed using the individual likelihoods of all cohort members [46]. These are the probabilities for survival throughout each member's follow-up period, multiplied—for cancer cases—by the probability for cancer occurring during the final year. Equivalently to maximizing L, we minimize the deviance, $\mathcal{D} = -2 \ln L > 0$, using the Minuit function-minimization library [47]. For model selection, we rely on the likelihood-ratio test for nested models so as to retain only parameters significant at a 95% confidence level. The same level is also adopted throughout this paper for confidence intervals. To rank non-nested models, the entropy-based Akaike index is used [48], $\text{AIC} = \mathcal{D} + 2n$, which effectively penalizes overfitting for models with larger number of parameters n. Without loss of information, we will denote only the difference ΔAIC with respect to the benchmark descriptive model.

Results

Table 1 provides an overview of the best-fitting models. Before explaining in detail their mechanisms and the implications for radiation risk, let us anticipate some general patterns. All highest-ranked multi-stage models share a Plutonium-induced enhancement of proliferation rates. More specifically, the fits suggest that 3-stage models with a radiation effect on an early stage of proliferation (models A, B) may yield an improved description of the Mayak data, compared with an effect on the penultimate stage (C). Although model A exhibits by far the lowest AIC, we will present the radiation risks for several three-stage models so as to give an impression of the range of model predictions, as discussed in the Appendix.

No evidence is found for a radiation-induced second pathway. We note that all dose rates here refer to internal Pu exposure; external radiation is not significant in the two-stage and descriptive models and thus not considered in the following.

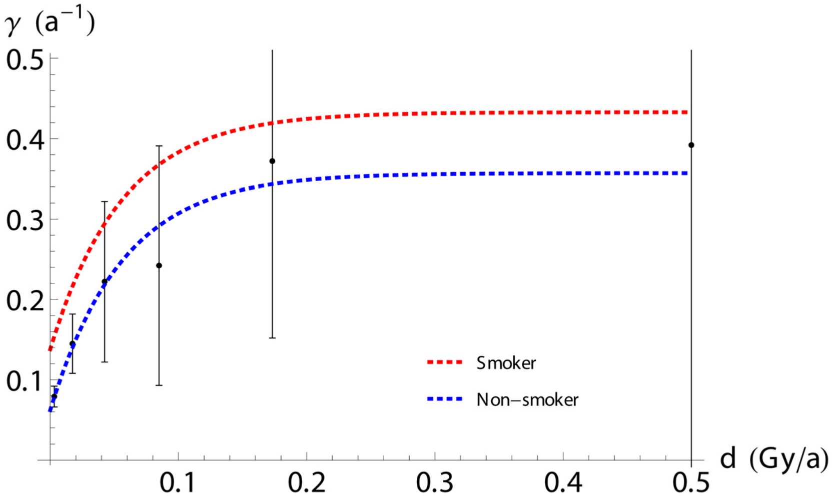

Fig 2. Dependence of the proliferation rate, $\gamma(d)$, on internal (lung) dose rate in the TSCE model. For comparison, the dots illustrate the results of a categorical fit (with 95%-level error bars).

doi:10.1371/journal.pone.0126238.g002

Two-stage model

The two-stage model has been applied to previous follow-ups of the Mayak workers [31, 32]. We shall therefore discuss it here as a benchmark, but also to illustrate some mechanisms inherent in any multistage model.

We find that the effects of both Pu dose rate as well as smoking status are by far best described as additively enhancing the net proliferation rate, $\gamma(s, d) = \gamma^{(0)}(s) + \delta\gamma(d)$. (An additional radiation effect on the initiating rate, $\mu_0(d)$, is highly insignificant when fitted to the data and thus not included in our model. This may be simply due to lack of data power and does not imply absence of radiation-induced initiating events.) A categorical fit of the dose dependence strongly suggests a saturation at larger dose rates (Fig 2), and we find it best modeled by an exponentially leveling function

$$\delta\gamma(d) = \gamma_\infty(1 - e^{-r\times d/\gamma_\infty}). \tag{2}$$

Here $r \sim 5$/Gy (see Table 3) governs the linear, low-dose response, $\delta\gamma \sim r\,d$, and $\gamma_\infty \sim 0.3$/a denotes the rate approached as $d \gg d_* \equiv \gamma_\infty/r$; here $d_* \sim 0.06$Gy/a. This is qualitatively in line with previous analyses [31, 32] but also several Radon-risk studies [23, 25, 27, 29, 30], see Discussion. It is worth noting that the data fit equally well to a response in terms of the accumulated dose D—i.e., $\delta\gamma(D)$ in Eq (2)—which may relate to the long protracted exposures of Mayak workers.

It should be stressed that, even though the main risk factors, smoking and Plutonium, enter the growth rate additively, the actual risk will exhibit an interaction between them. To illustrate this, in Fig 3(a) we display the age-dependent hazard, $h(t)$, for a representative exposure scenario at a constant dose rate from age t_1 = 25a to 60a, with dose D = 0.2Gy. For a wide age range, coinciding with the phase of exponential proliferation, the relative risks of radiation and smoking approximately multiply (note the log scale). It is only at larger ages ($\gtrsim$ 60a) that the combined risk drops below the multiplicative value. Such sub-multiplicity agrees with trends glimpsed in a descriptive analysis [37]. Here, it follows naturally because the hazard of Pu-exposed smokers levels off much earlier, reflecting an earlier onset of cancer (see Methods).

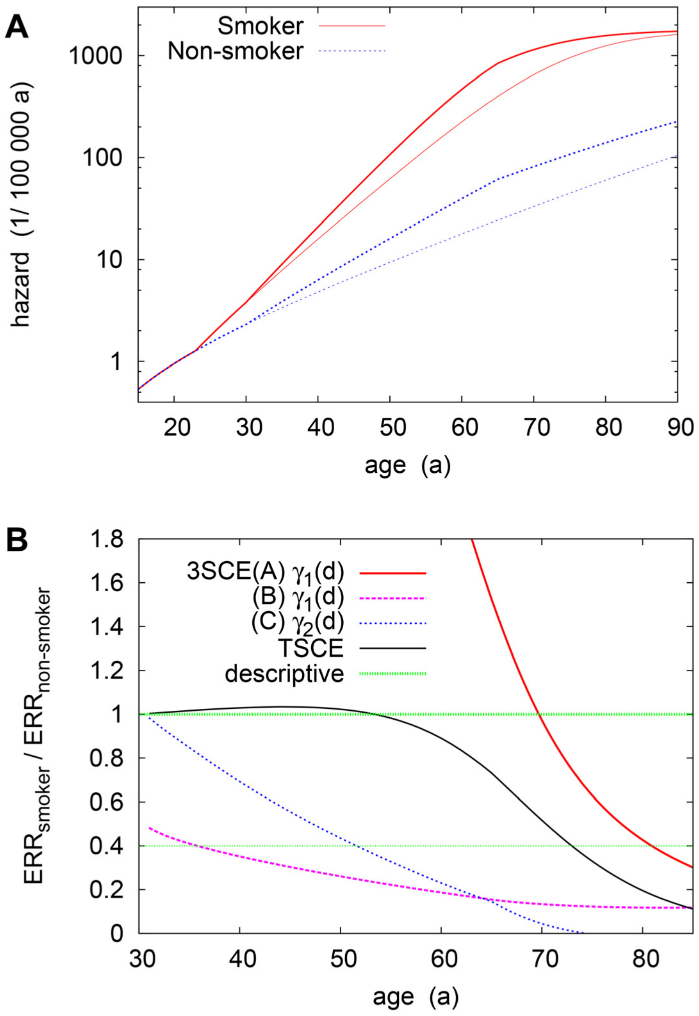

Fig 3. Risk for a scenario with constant exposure between ages 25 and 60, at D = 0.2Gy. Notice the lag time of 5a. (a) Hazard $h(t)$ of the two-stage model for non-/smokers. Shown are values without (baseline) and with exposure. (Smoking is assumed to start at age 18.) (b) Excess-relative-risk (ERR) ratio of smokers and non-smokers, shown for different multi-stage models (see text). A ratio of 1 indicates multiplicative Plutonium-smoking interaction, as in the standard descriptive model. The thin-dotted line denotes the (non-significant) sub-multiplicative trend suggested by an extended descriptive model.

doi:10.1371/journal.pone.0126238.g003

To better link these findings to those of descriptive models (see Appendix), we will from now on consider the excess relative risk, ERR $\equiv h/h_{bsl} - 1$, defined relative to the zero-exposure baseline risk. From this angle, multiplicity of risk implies an equal ERR for smokers and non-smokers—i.e., a ratio $ERR^{(s=1)}/ERR^{(s=0)} = 1$. Fig 3(b) shows that, under the scenario above, the risk is indeed multiplicative until older ages ($t \lesssim$ 60a). It then becomes sub-multiplicative, and the ERR ratio drops markedly below unity after (time-lagged) exposure ends.

Since most cases are related to smoking, we will now concentrate on the risk for smokers. Moreover, to separate age and dose dependencies, we scale the ERR by the accumulated dose, $ERR(D;t)/D(t - t_{lag})$, with the lag time t_{lag} = 5a. Fig 4 displays the age-dependent ERR/D for the scenario above but at D = 0.5Gy. For the two-stage model, it reveals a characteristic increase during exposure (owing to proliferation), followed by a marked drop-off after exposure has

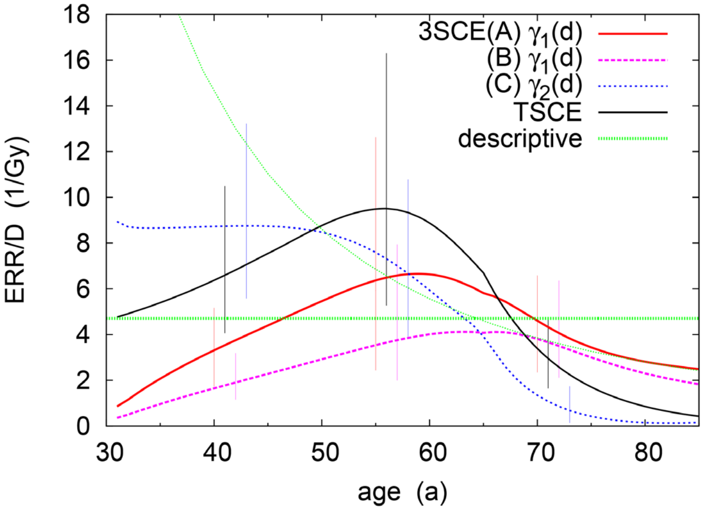

Fig 4. Age-dependent excess relative risk (ERR/*D*) of different multi-stage models, for smokers with constant exposure between ages 25 and 60 (*D* = 0.5Gy). For comparison, the non-significant trend in the descriptive model (thin-dotted line) is also shown. (All error bars are at 95% confidence level.)

doi:10.1371/journal.pone.0126238.g004

ended. The latter is similar to the (non-significant) attained-age trend found in the descriptive model, cf. Appendix.

The age dependence just discussed pertains to one specific scenario. Let us now indicate how the risk is modified by different exposure patterns. The dependence upon age at first exposure, t_1, is shown in Fig 5(a). We have chosen a scenario with $D = 0.2$Gy and a typical duration $\Delta t = 20$a; the ERR is recorded at $t_1 + \Delta t + t_{lag}$. Clearly, for this two-stage model, the variation is rather mild for all but very early exposures (not encountered at Mayak) and very late ones. In the former case, virtually no premalignant cells are available for proliferation. At older ages, in turn, they are increasingly lost to new malignancies; thus the risk is attenuated. In the descriptive model, a weak (non-significant) trend also suggests a slight decrease of ERR with older ages at exposure (not shown).

Fig 5(b) reveals a characteristic influence of exposure duration, Δt (where $D = 0.2$Gy, $t_1 =$ 20a). For very short exposures, $\Delta t \ll D/d_* \sim 3$a, the ERR is strongly suppressed: The short duration cannot be compensated by an increased dose rate, since the growth rate saturates at dose rates larger than d_*. This inverse dose-rate effect is thus inherently connected to a saturating radiation response as in Eq (2). It has been observed also in mechanistic Radon studies [23, 25, 30], although the Mayak data are not powerful enough to support this at the descriptive level. At sufficiently long exposures, $\Delta t \gg D/d_*$, the inverse dose-rate effect disappears and eventually gives way to a slight direct effect. This is related to the leveling of the hazard upon reaching malignancy.

To wrap up this discussion, let us consider the dose-response relationship implied by this proliferation-based model. Fig 6 illustrates that, in contrast to the linear dose response typically assumed in descriptive modeling ($\mathrm{ERR}(D) = cD$, here $c \approx 4.7$/Gy), this model exhibits a nonlinear response. Although the quantitative values depend on the exposure scenario (here: exposure during $t = 25 - 60$a), some general features apply to any two-stage model with a proliferation enhancement similar to Eq (2): The dose response is characterized by a linear low-dose regime, $\mathrm{ERR} \simeq r \times D$ for $D \ll 1/r$, and leveling at sufficiently large dose(rate)s and/or ages. Intermittently, typically an exponential increase is seen, reflecting the exponential growth

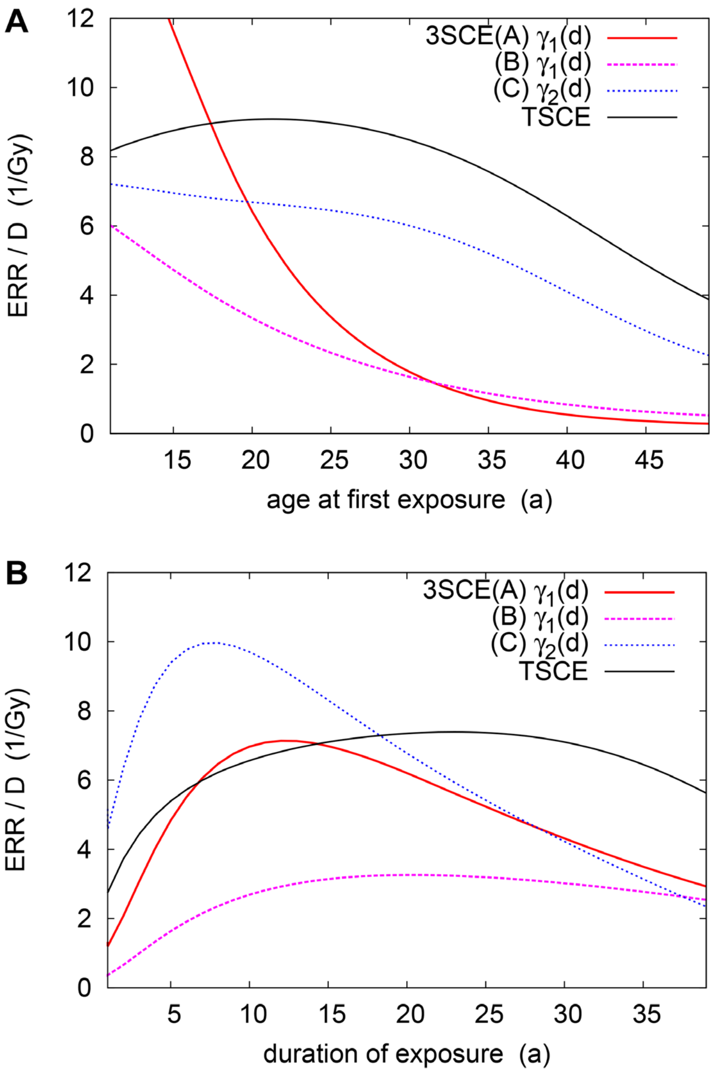

Fig 5. Dependence of ERR/*D* on different exposure patterns, for smokers with *D* = 0.2Gy. (a) ERR/*D* for different ages at first exposure, t_1 (at fixed duration Δt = 20a). (b) ERR/*D* for different durations Δt (at fixed t_1 = 20a).

doi:10.1371/journal.pone.0126238.g005

of premalignant cells due to proliferation. However, this effect tends to be washed out by the leveling at older ages. In a descriptive model fit, where essentially an averaging occurs over all exposure histories in the cohort, this pronounced nonlinearity will be even harder to resolve. Still, it is noteworthy that a TSCE-inspired descriptive model, ERR = $e^{f(D)} - 1$ with $f(D) \equiv c_\infty(1 - e^{-c_{\rm lin}D/c_\infty})$, yields an improved fit (but similar AIC), with a linear response $c_{\rm lin} \sim 3.2$/Gy and leveling at $D_* \equiv c_\infty/c_{\rm lin} \sim 0.8$Gy.

Three-stage models

As mentioned earlier, the highest-ranked 3-stage models fall into two categories.

The best two models (A, B; see Table 4) show a radiation effect on the *earlier* stage of proliferation, $\gamma_1(d)$, differing only in their background parameters—specifically the smoking

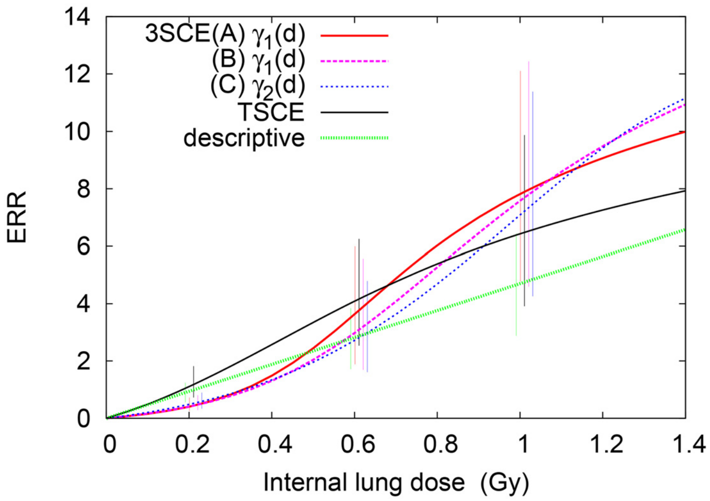

Fig 6. Dose-response relationship, ERR(*D*), for exposure during ages 25 to 60, recorded at age 65. For comparison, the linear response ERR = *cD* is plotted.

doi:10.1371/journal.pone.0126238.g006

response on μ_1 (A) or γ_2 (B). This early impact leads to a substantially delayed radiation response compared to both the 2-stage model and 3-stage model C, as is seen from Fig 4: The ERR/D is initially zero and, once it has peaked, tends to drop off more mildly. (In some scenarios, the ERR/D may even increase when exposure stops.) This lag is intuitive, as the insulted cells need to pass through an additional mutation stage before becoming malignant. In stark contrast, model C—with an effect on the penultimate stage—predicts a much higher risk, ERR/$D \simeq r \sim 10$/Gy, right after exposure. In the scenario depicted in Fig 4, it further displays an almost monotonic drop with attained age, not unlike the trend seen in the descriptive model risk.

However, it must be stressed that this specific scenario conceals an underlying complexity not present in the 2-stage model. The reason is that the 3-stage model is determined by two competing growth rates, γ_1 and γ_2. Let us consider models A/B: For a small enough dose rate such that $\gamma_1^{(0)} + rd < \gamma_2^{(0)}$, the clonal dynamics is governed by the largest baseline rate, $\gamma_2^{(0)}$. In other words, below the critical dose rate $d_{\text{crit}} \equiv (\gamma_2^{(0)} - \gamma_1^{(0)})/r \sim 0.01\text{Gy/a}$, both baseline and excess risk grow with the same exponential rate—hence the ERR would level off with age. It is only above that critical dose rate that an exponential increase is seen in the ERR. Likewise, for model C, the critical point, $(\gamma_1^{(0)} - \gamma_2^{(0)})/r$, marks the dividing line between exponential increase and leveling with age. Notice that the value in Fig 4 is just at the borderline.

The dose-rate dependence just described is reflected in the dose response (Fig 6). At low doses, a linear increase is seen, just as for the two-stage model. However, that low-dose response is typically much lower than what we saw for two-stage and descriptive models. It is only at the critical dose, here $d_{\text{crit}}\Delta t \sim 0.35$Gy, that a rapid exponential increase sets in. Compared to the two-stage case, this exponential increase is much sharper owing to the higher response coefficients, $r \sim (10 - 17)$/Gy. As before, the response levels off once the corresponding dose rates exceed $d_* \sim (0.03 - 0.05)$Gy/a.

Another consequence of an early-stage radiation effect is a notably suppressed risk for older ages at exposure, t_1 [Fig 5(a)]. To understand this, recall that the radiation effect consists in multiplying the available pool of stage-1 cells, $X_1(t_1)$, prior to their making the transition to

stage 2. Since that number grows only very slowly at a rate $\gamma_1^{(0)} < \gamma_2^{(0)}$, the head start given to already existing stage-2 cells, $X_2(t_1)$, becomes overwhelming the later in life exposure starts. Thus the ERR is suppressed exponentially as $e^{-(\gamma_2^{(0)}-\gamma_1^{(0)})t_1}$ for large t_1. By contrast, the mechanism for model C is fairly similar to that of the 2-stage model: Both radiation and baseline risk are governed by the growth of existing stage-2 cells. This is why virtually no dependence on t_1 is seen other than a mild drop-off for older ages at exposure, due to the onset of malignancies.

Differences with respect to the two-stage model may also be observed in the exposure-duration dependence [Fig 5(b)]. Like the 2-stage models, all 3-stage models exhibit an inverse dose-rate effect for durations $\Delta t \lesssim D/d_*$. As explained previously, this merely hinges on the saturation of the proliferation rate for higher dose rates. It may be noted that the early-stage models A/B lead to a stronger risk suppression at such short durations, due to the extra mutational step to be passed. However, a more qualitative difference is found for longer durations: Here a marked direct effect occurs, the ERR falling off as Δt^{-1}. This may be viewed as a linear dose-rate modification for perturbatively small dose rates $D/\Delta t$ in the limit of long durations.

Finally, let us comment on the interaction between radiation and smoking risks. We saw earlier that the two-stage model predicts a largely multiplicative interaction or, equivalently, a near-unit ratio of smokers' and non-smokers' ERR [Fig 3(b)]. The situation is less clear cut for the three-stage models. Model C, with a later-stage radiation effect, is most comparable to the 2-stage case: Initially, it also exhibits near-multiplicity, which reflects that radiation simply leads to multiplication of existing stage-2 cells. However, the ERR ratio then drops very rapidly. This is essentially because for smokers, the dose rate is *below* the critical value, $(\gamma_1^{(0)} - \gamma_2^{(0)})/r \sim 0.01\mathrm{Gy/a}$, as smoking leads to a very large growth rate $\gamma_1(s = 1) \approx 0.16\mathrm{a}^{-1}$. Thus the ERR levels off, in contrast to the exponentially growing ERR for non-smokers, where there is no threshold. An analogous mechanism is at work in the model B, which is sub-multiplicative throughout.

By contrast, for model A, the risk in the scenario shown in Fig 3(b) is strikingly super-multiplicative except for ages $t \gtrsim 70\mathrm{a}$. At first glance, this deviation from multiplicity might seem surprising: For constant rates, the hazard is typically proportional to $\mu_1(s)$, and the smoking dependence should thus drop out of the relative risk. However, smoking is assumed to start at age 18, only a few years prior to irradiation. Hence the baseline risk—initially proportional to the number of existing stage-2 cells—mostly stems from those cells created *before* smoking started, and is thus comparable for non-/smokers. Thus, the large ERR for smokers reflects the much higher excess risk due to freshly mutated stage-1 cells. It is only long after the start of exposure that the smokers' baseline risk increases sufficiently to compensate for this.

Two-pathway models

We have tested a family of multi-path models of genomic instability (GI). Let us briefly outline the key assumptions made here to reduce the number of parameters—in total, 6 mutation rates and 6 cell-division/death rates, as sketched in Fig 1(bottom). Most premises are motivated by the biological mechanisms thought to underlie GI [14, 44]. First, the destabilizing transition rates are set equal, $\sigma_j \equiv \sigma$, as they pertain to the inactivation of the same genomic-integrity gene. Since, presumably, GI *per se* does not lead to any growth advantage, we set the birth/death rates at stage 0^{GI} equal, $\alpha_0^{\mathrm{GI}} = \beta_0^{\mathrm{GI}}$, their precise value being marginal. Mutation rates following GI, μ_j^{GI}, are supposed to be larger, or at least equal, compared to their genomically stable (upper-branch) counterparts.

Although GI may well be present as a sporadic mechanism, the baseline data are not thought to provide sufficient structure to distinguish complex background models (see

Discussion; cf. also Ref. [15]). We have thus focused on the case of radiation-induced GI—i.e., an activation of the otherwise silent GI path (σ) by radiation. This may be modeled as $\sigma = r_{GI} \times d$ (with vanishing background rate). In addition, radiation may affect regular mutation or growth rates.

None of the tested two-pathway models has led to any significant, numerically stable improvement beyond the benchmark two-stage model. It is stressed that relaxing the assumptions of linearity or vanishing background rate do not yield a qualitative improvement.

Discussion

Mechanism of radiation action

A robust result of our analysis is an α-radiation-induced enhancement of proliferation rates of premalignant cells. This corroborates a consistent finding in many studies based on fitting two-stage models to lung-tumor data, both from other epidemiological cohorts (Radon-exposed miners [23, 25, 27, 29, 30]) and animal experiments (see Ref. [49] for an overview). It also alleviates concerns that a proliferation effect might be confined to the two-stage model [34].

However, it has long been criticized [33] that no radiobiological evidence exists for such a premalignant-growth-enhancing effect. Although experimental evidence is still sparse [50, 51], let us briefly discuss some theoretical models put forward to explain how radiation might lead to enhanced proliferation of premalignant cells.

The most elaborate model is based on the following idea [52]: Radiation kills cells, which in turn triggers division of neighboring stem cells so as to replenish the lost tissue. This may lead to net proliferation of premalignant cells if these have a selective advantage, that is, a slightly higher rate for cell division than needed for homeostasis. (Strictly speaking, cells need not necessarily be killed; it might be sufficient for them to have a proliferative disadvantage—e.g., by effected cell-cycle arrest—such that these are subsequently repelled by premalignant cells.) That hypothesis has been shown [53–55] to lead to a dose-rate response, $\gamma(d)$, which is qualitatively similar to that found in cohort studies (see, e.g., Fig 2), albeit with quantitatively modest agreement. Saturation of the growth rate much higher than a characteristic dose rate, $d \gg d_s$, occurs if more cells are killed than can be substituted for by premalignant cells.

To obtain a very rough estimate for that critical dose rate, note that α-particle hits of a cell nucleus are independent and rare. Thus the number of hits, N, is Poisson-distributed, $P_N = e^{-\bar{N}}\bar{N}^N/N!$, which means the fraction of cells *not* hit is $P_{N=0} = e^{-\bar{N}}$. Assuming (i) a linear dose response, $\bar{N} = n\,D$, with $n \sim 4/\mathrm{Gy}$ [56], and (ii) delivery of the dose $D \equiv d\,\tau$ over a characteristic time of order the interval between cell cycles ($\tau \sim 1\mathrm{a}$ for basal stem cells [57]), we have $P_{N=0}(d) = e^{-nd\tau}$. At the characteristic dose rate, d_s, about one out of, say, six nearest neighbors would be hit, such that $P_0(d_s) = 1 - p$, $p \equiv 1/6$, yielding a characteristic dose rate of

$$d_s \simeq \frac{p}{n\tau} \sim 0.04\frac{Gy}{\mathrm{a}}. \qquad (3)$$

This is on the same order of magnitude as the value found in this study, $d_* \sim (0.03 - 0.06)\mathrm{Gy/a}$. In this light, the model results presented here and the repopulation mechanism may be interpreted to be compatible. However, it is important bearing in mind that these estimates are naturally crude. Matters are further complicated by the spatially inhomogeneous energy deposition within different spots in the lungs, an effect not reflected in whole-lung doses used here [58].

As an alternative mechanism, a radiation-induced disturbance of cell communication has been suggested [9, 59]. This may lead to, e.g., up-regulated growth signals or a reduction of apoptosis [60, 61], with a higher effect on intermediate cells because those tend to evade

homeostatic control. It has even been proposed that a proliferation enhancement, mediated by such a bystander signaling, might be the generic mechanism for the response to densely ionizing radiation [62]. However, no mechanistic model has been put forward explaining in detail how this might lead to a dose-rate response, $\gamma(d)$.

Even so, should the radiation response indeed be governed by the bystander effect, then a similar behavior ought to be expected as for bystander-mediated mutation induction [63]. In microbeam experiments [64, 65], it has been found that for low doses—corresponding to less than $\sim$ 10% of cells being hit—the mutagenic yield was strongly amplified as bystander cells also received signals. (A similar pattern has been found for intercellular induction of apoptosis [66].) For much higher doses, in turn, the response essentially saturated. Along the lines leading to Eq (3), we can estimate the characteristic dose rate d_s by assuming the crossover to occur at a fraction $p = 0.1$ of cells being hit. This yields $d_s \approx p/n\tau \sim 0.025$Gy/a, under the same caveats as mentioned above. From this standpoint, both bystander signaling and the repopulation hypothesis appear compatible with the dose-rate response found in this study.

Comparison with previous studies

As discussed earlier on, for mechanistic models, the risk is essentially determined by its structure, particularly, the radiation response. A dose-rate dependent proliferation rate, $\gamma(d)$, saturating for larger dose rates (as in Eq 2) is found in many studies applying the two-stage model to α-particle-induced lung cancer. In particular, our response quantitatively agrees with that of the preferred two-stage model by Jacob *et al.* for the Mayak cohort, both for Plutonium and smoking [32]. Concordantly, their risk estimates are similar to those of the present TSCE model—such as a cohort-averaged excess risk of $\mathrm{ERR}(t = 60\mathrm{a})/D \sim 4/\mathrm{Gy}$, a nonlinear dose dependence ERR(D) for larger doses, and sub-multiplicity of smoking and radiation risks.

In a recent descriptive analysis of the Mayak data, Gilbert *et al.* found a linear dose response, modified significantly only by a drop-off with attained age [37], see also Appendix. The value at age 60, $\mathrm{ERR}(t = 60\mathrm{a}) \sim 7D/\mathrm{Gy}$, is somewhat higher than for the cohort average of the mechanistic models presented here. By contrast, the multi-stage models exhibit a strongly nonlinear dose dependence especially for higher doses. Furthermore, they typically display a decrease with attained age only for large enough ages, most pronounced after the end of exposure. Initially, an increase with age is seen due to exponential clonal growth, at least for high enough doses.

In contrast to descriptive models, where an exposure modifier may not be significant when parametrized explicitly, mechanistic models implicitly make predictions for the risk dependence on any exposure scenario. This is exemplified by the age-at-exposure dependence or the inverse dose-rate effect shown by several mechanistic models (Fig 5). Furthermore, a non-significant trend in Ref. [37] indicated a sub-multiplicative interaction between Plutonium dose and smoking. This is in agreement with results from the 2-stage model, which further offers a mechanistic interpretation in terms of exponential cell growth, combined with earlier malignancies for smokers (see Results).

Implications for lung carcinogenesis

We have shown that several three-stage models give an improved description of the data compared to one involving two stages. Evidently, this stochastic inference is based solely on the lung-cancer endpoint and cannot replace experimental insight into the dynamics of intermediate stages. Even so, it is worth emphasizing that the Mayak data do not fully allow to distinguish general mechanisms for carcinogenesis. Rather, the results here mostly rely on the radiation-associated risk. In fact, the deviances of the various mechanistic and descriptive

Table 2. Observed numbers of lung-cancer cases by dose categories, compared with those predicted by the descriptive and multi-stage models (in brackets: excess cases). As a reference, we also give the person years (py), i.e., the number of years spent in the respective (sub)cohort summed over all persons.

Doses (Gy)	py	Cases	TSCE	3SCE(A)	3SCE(B)	3SCE(C)	descriptive
0 – 0.01	188,995	204	198 (0.5)	203 (0.2)	205 (0.2)	204 (0.3)	194 (0.6)
0.01 – 0.03	18,888	32	50 (3)	49 (1)	50 (1)	50 (1)	49 (4)
0.03 – 0.1	15,843	58	51 (9)	47 (4)	47 (4)	48 (4)	52 (11)
0.1 – 0.3	7,091	41	37 (15)	31 (8)	30 (23)	31 (7)	39 (17)
0.3 – 1	3,153	29	33 (24)	34 (25)	31 (21)	31 (21)	32 (23)
> 1	738	24	18 (16)	24 (21)	25 (22)	25 (22)	22 (20)
total	234,708	388	388 (67)	388 (59)	388 (56)	388 (56)	388 (75)

doi:10.1371/journal.pone.0126238.t002

baseline models (risk factors age and smoking) do not differ noticeably—in contrast to the models including radiation (Table 1).

That statement seemingly contradicts the fact that there is only a fraction $\sim$ 25% of excess cases relative to the baseline (as can be seen from $\overline{\mathrm{ERR}} \sim 5\bar{D}/\mathrm{Gy}$, the whole-cohort average dose being $\bar{D} \sim 0.05\mathrm{Gy}$). This translates to 60–70 excess cases (Table 2), compared to about 320 baseline cases. However, truly spontaneous baseline cases (30–40) are outnumbered by smoking-related ones by a factor of $\sim$ 10. Moreover, the smoking variable is only binary (and noisy). This makes it hard to resolve the actual baseline risk accurately, especially since the multi-stage models do not differ in their qualitative behavior except for young ages. By contrast, the models do differ markedly for different irradiation scenarios as found in the Mayak data.

Conclusion

We have shown that carcinogenesis models extended to three stages offer an improved description of the Mayak lung-cancer data, as compared to the two-stage model. All favored 3- and 2-stage models indicate a radiation-enhanced proliferation rate of premalignant cells, suggesting that this is a robust finding—at least in the framework of multi-stage models with clonal expansion— and not limited to the case of two stages. Despite that structural similarity, the models make qualitatively different predictions for the risk following certain exposure scenarios. For instance, those models whose radiation impact is on an earlier stage exhibit a strongly suppressed risk for older ages at exposure. As opposed to the two-stage case, all three-stage models reveal a critical dose(rate) above which the excess risk increases sharply. Moreover, while an inverse dose-rate effect is predicted by all models, only those with three stages also display a pronounced direct effect for longer exposure durations.

One aim of this study has been to elucidate which aspects of carcinogenesis models are persistent themes or rather model-specific features. Such a better understanding should facilitate the development of improved carcinogenesis models, both mechanistic but also descriptive ones. There is still some way to go toward a more realistic description. Ultimately, this would involve biological input on rates or premalignant stages so as to cut the number of undetermined parameters. Closer at hand, a natural next step may be validating the current models on other data sets. An extension to more than three stages conceivably provides a further improvement. Moreover, to get a more accurate description of the biological mechanisms, it is desirable to develop models specifically for the different histological cancer subtypes.

Table 3. Key parameter estimates for the two-stage model, including 95%-level uncertainties. For definitions and interpretation of the parameters used, see text. (Note that only certain rate combinations can be determined from the hazard.)

Parameter	TSCE
$N\mu_0\,\mu_1$ (a^{-2})	$(3.9^{+5.1}_{-2.4}) \times 10^{-7}$
$\alpha\mu_1$ (a^{-2})	$(3.0^{+2.7}_{-1.5}) \times 10^{-6}$
γ (a^{-1})	$0.062^{+0.024}_{-0.025}$
$\Delta\gamma(s)$ (a^{-1})	$0.076^{+0.020}_{-0.017}$
r (Gy^{-1})	$5.2^{+2.7}_{-1.7}$
γ_∞ (a^{-1})	$0.30^{+0.35}_{-0.12}$

doi:10.1371/journal.pone.0126238.t003

Appendix: Parameter estimates

We now present some details on the model results.

The reference descriptive model closely follows Ref. [37]. The main risk factor is smoking, which is included simply as a factor in Eq (1), $e^{\psi_{\rm smk}} = e^{2.3\pm0.3} \approx 10$. The alcohol status further increases the baseline risk by $e^{\psi_{\rm alc}} = e^{0.6\pm0.1} \approx 1.8$. In addition, the birth year was found to elevate the risk between years 1915 and 1935 by about $e^{0.4\pm0.1} \approx 1.5$. With 3 extra parameters, this birth-cohort effect is moderately significant ($\Delta\mathcal{D} = -11$). It might be interpreted as a smoking modifier (as which it yields only a slightly higher deviance), possibly related to the changed smoking levels between the wars. The effect is irrelevant for the radiation risk, and generally no birth-cohort distinction is made in the scenarios in Figs 3–6. We mention that a calendar-year dependence has been tested but discarded: It oscillates strongly but shows no conclusive trend, nor does it influence the dose response.

The dose dependence is modeled as a linear function, ERR = cD, $c = (4.7 \pm 0.9)\mathrm{Gy}^{-1}$, with no evidence for threshold or quadratic terms. Note that the lag time entering the dose, $D \equiv D(t - t_{\rm lag})$, does not alter the deviance significantly between 0–10a. We fix it at $t_{\rm lag}$ = 5a, also for all multi-stage models. No significant time-dependent dose modifiers are found. However, a trend suggests a decrease of ERR/D with attained age, modeled as $(t/60\mathrm{a})^{-2.4\pm2.5}$, and a marginal decrease with median age at exposure. Even though the reference model (Eq 1) implies multiplicative risks of smoking and radiation, a non-significant trend indicates sub-multiplicity, with an ERR for smokers reduced by a factor $e^{-0.9(\pm1.5)} \sim 0.4$.

The maximum-likelihood parameters of the TSCE model are shown in Table 3. We have omitted two covariables not relevant for the radiation risk: alcohol and birth year. Both are included as addends to the growth rate γ, consistent with an interpretation as smoking surrogates. We have also tested an explicit age dependence of the rate parameters, which indicated a reduction of $\mu_1(t)$ or, equivalently, $\gamma(t)$ above age $\sim$ 50a. However, these effects were numerically unstable and have thus been discarded. To convey an impression of the fit quality, Table 2 shows a juxtaposition of observed cancer cases and those predicted by the various models across the dose range.

A comment is in order on the parameter estimates of the three-stage models (Table 4). Some of the (structurally) identifiable parameter combinations may be practically indeterminable insofar as they leave the minimum deviance $\mathcal{D}$ virtually unchanged. In model A, for instance, $\mathcal{D}$ is independent of $\mu_1\,\mu_2 \to 0$, which has been fixed at an arbitrarily small value. Furthermore, the best estimate of $\gamma_1^{(0)} = \alpha_1 - \beta_1 \approx -0.1\mathrm{a}^{-1}$ is not significant, and $\gamma_1^{(0)}$ is set to zero. Worse yet, for model B (C), the background rate $\gamma_2^{(0)}$ ($\gamma_1^{(0)}$) is unstable, tending to

Table 4. Same as Table 3, but for the parameters of the best three-stage models.

Parameter	3SCE(A)	3SCE(B)	3SCE(C)
$N\mu_0\ \mu_1\ \mu_2$ (a^{-3})	$(1.3^{+2.6}_{-0.9}) \times 10^{-9}$	$(2.0^{+3.8}_{-1.8}) \times 10^{-8}$	$(4.0^{+6.9}_{-2.6}) \times 10^{-8}$
$\alpha_1\ \mu_1\ \mu_2$ (a^{-3})	$(1.0^{+1.6}_{-0.6}) \times 10^{-8}$	$(1.2^{+2.8}_{-1.1}) \times 10^{-7}$	$(4.7^{+4.8}_{-2.4}) \times 10^{-7}$
$\alpha_2\ \mu_2$ (a^{-2})	$(8.4^{+11}_{-5.5}) \times 10^{-6}$	$(0.7^{+6.7}_{-0.6}) \times 10^{-5}$	+0[a]
$\mu_1\ \mu_2$ (a^{-2})	+0[a]	+0[a]	$(1.2^{+2.4}_{-0.8}) \times 10^{-6}$
$\alpha_1 - \beta_1$ (a^{-1})	0 (n.s.)[b]	$0.068^{+0.062}_{-0.048}$	0 (n.s.)[b]
γ_2 (a^{-1})	0.16 ± 0.03	0 (n.s.)[b]	$0.050^{+0.035}_{-0.056}$
(smoking)	$\mu_1 \times e^{3.6\pm0.8}$	$\gamma_2 + ((0.14^{+0.13}_{-0.02}))a^{-1}$	$\gamma_1 + (0.16 \pm 0.03)a^{-1}$
r (Gy^{-1})	17^{+9}_{-5}	10^{+6}_{-4}	10^{+6}_{-3}
γ_∞ (a^{-1})	$0.54^{+0.18}_{-0.13}$	$0.46^{+0.19}_{-0.13}$	$0.39^{+0.19}_{-0.13}$

[a] Parameter not converged, set to arbitrarily small, positive value ($\sim 10^{-10}$).

[b] Not significant; set to zero.

doi:10.1371/journal.pone.0126238.t004

unrealistically large negative values. Since only the smokers' value is stable, $\gamma_2^{(0)} + \Delta\gamma_2(s = 1) \sim 0.1a^{-1}$, we have set $\gamma_2^{(0)} \equiv 0$, even at the price of a higher deviance. It is also for these intricacies that we have opted to present several of the best three-stage models, rather than relying strictly on the weight suggested by Akaike's index. This way, a more plausible impression is given of the uncertainty of model predictions.

Acknowledgments

S.Z. thanks R. Meckbach, C. Simonetto, P. Kundrat, B.G. Madas, M. Rosemann, P. Jacob, J.C. Kaiser, and M. Blettner for fruitful discussions. This work was supported by the European Commission under FP7 project no. 269553 (EpiRadBio).

Author Contributions

Conceived and designed the experiments: MS. Performed the experiments: MS. Analyzed the data: SZ. Contributed reagents/materials/analysis tools: ME SZ. Wrote the paper: SZ.

References

1. Weinberg R (2013) The Biology of Cancer. Garland Science.
2. Hanahan D, Weinberg RA (2011) Hallmarks of cancer: the next generation. Cell 144: 646–74. doi: 10.1016/j.cell.2011.02.013 PMID: 21376230
3. Nordling C (1953) A new theory on the cancer-inducing mechanism. Br J Canc 7: 68. doi: 10.1038/bjc.1953.8
4. Armitage P, Doll R (1954) The age distribution of cancer and a multi-stage theory of carcinogenesis. Br J Canc 8: 1. doi: 10.1038/bjc.1954.1
5. Knudson AG (1971) Mutation and cancer: statistical study of retinoblastoma. PNAS 68: 820–3. doi: 10.1073/pnas.68.4.820 PMID: 5279523
6. Moolgavkar SH, Venzon DJ (1979) Two-event models for carcinogenesis: incidence curves for childhood and adult tumors. Math Biosci 47: 55–77. doi: 10.1016/0025-5564(79)90005-1
7. Tan WY (2008) Handbook of cancer models with applications, volume 9. World Scientific.
8. Little MP (2010) Cancer models, genomic instability and somatic cellular Darwinian evolution. Biol Direct 5: 19; discussion 19 doi: 10.1186/1745-6150-5-19 PMID: 20406436

9. Jacob P, Meckbach R, Kaiser JC, Sokolnikov M (2010) Possible expressions of radiation-induced genomic instability, bystander effects or low-dose hypersensitivity in cancer epidemiology. Mutat Res 687: 34–9. doi: 10.1016/j.mrfmmm.2010.01.005 PMID: 20096708
10. Little MP, Wright EG (2003) A stochastic carcinogenesis model incorporating genomic instability fitted to colon cancer data. Math Biosci 183: 111–134. doi: 10.1016/S0025-5564(03)00040-3 PMID: 12711407
11. Little MP (1995) Are two mutations sufficient to cause cancer? Some generalizations of the two-mutation model of carcinogenesis of Moolgavkar, Venzon, and Knudson, and of the multistage model of Armitage and Doll. Biometrics 51: 1278. doi: 10.2307/2533259 PMID: 8589222
12. Luebeck EG, Moolgavkar SH (2002) Multistage carcinogenesis and the incidence of colorectal cancer. PNAS 99: 15095–100. doi: 10.1073/pnas.222118199 PMID: 12415112
13. Lengauer C, Kinzler KW, Vogelstein B (1997) Genetic instability in colorectal cancers. Nature 386: 623–627. doi: 10.1038/386623a0 PMID: 9121588
14. Nowak MA, Komarova NL, Sengupta A, Jallepalli PV, Shih IM, Vogelstein B, et al. (2002) The role of chromosomal instability in tumor initiation. PNAS 99: 16226–31. doi: 10.1073/pnas.202617399 PMID: 12446840
15. Little MP, Li G (2007) Stochastic modelling of colon cancer: is there a role for genomic instability? Carcinogenesis 28: 479–87. doi: 10.1093/carcin/bgl173 PMID: 16973671
16. Little MP, Vineis P, Li G (2008) A stochastic carcinogenesis model incorporating multiple types of genomic instability fitted to colon cancer data. J Theor Biol 254: 229–38. doi: 10.1016/j.jtbi.2008.05.027 PMID: 18640693
17. Kaiser JC, Meckbach R, Jacob P (2014) Genomic instability and radiation risk in molecular pathways to colon cancer. PLoS ONE 9: e111024. doi: 10.1371/journal.pone.0111024 PMID: 25356998
18. Meza R, Jeon J, Moolgavkar SH, Luebeck EG (2008) Age-specific incidence of cancer: Phases, transitions, and biological implications. PNAS 105: 16284–9. doi: 10.1073/pnas.0801151105 PMID: 18936480
19. Luebeck EG, Curtius K, Jeon J, Hazelton WD (2013) Impact of tumor progression on cancer incidence curves. Cancer Res 73: 1086–1096. doi: 10.1158/0008-5472.CAN-12-2198 PMID: 23054397
20. Hazelton WD, Clements MS, Moolgavkar SH (2005) Multistage carcinogenesis and lung cancer mortality in three cohorts. Cancer Epidemiol Biomarkers Prev 14: 1171–81. doi: 10.1158/1055-9965.EPI-04-0756 PMID: 15894668
21. Schöllnberger H, Manuguerra M, Bijwaard H, Boshuizen H, Altenburg HP, Rispens SM, et al. (2006) Analysis of epidemiological cohort data on smoking effects and lung cancer with a multistage cancer model. Carcinogenesis 27: 1432–44. doi: 10.1093/carcin/bgi345 PMID: 16410261
22. Meza R, Hazelton WD, Colditz GA, Moolgavkar SH (2008) Analysis of lung cancer incidence in the nurses' health and the health professionals' follow-up studies using a multistage carcinogenesis model. Cancer Causes Control 19: 317–328. doi: 10.1007/s10552-007-9094-5 PMID: 18058248
23. Luebeck E, Heidenreich W (1999) Biologically based analysis of the data for the Colorado uranium miners cohort: age, dose and dose-rate effects. Radiat Res 351: 339–351. doi: 10.2307/3580219
24. Leenhouts HP (1999) Radon-induced lung cancer in smokers and non-smokers: risk implications using a two-mutation carcinogenesis model. Radiat Environ Biophys 38: 57–71. doi: 10.1007/s004110050138 PMID: 10384956
25. Hazelton WD, Luebeck EG, Heidenreich WF, Moolgavkar SH (2001) Analysis of a historical cohort of chinese tin miners with arsenic, radon, cigarette smoke, and pipe smoke exposures using the biologically based two-stage clonal expansion model. Radiat Res 156: 78–94. doi: 10.1667/0033-7587(2001)156%5B0078:AOAHCO%5D2.0.CO;2 PMID: 11418076
26. Brugmans MJP, Rispens SM, Bijwaard H, Laurier D, Rogel A, Tomásek L, et al. (2004) Radon-induced lung cancer in French and Czech miner cohorts described with a two-mutation cancer model. Radiat Environ Biophys 43: 153–63. doi: 10.1007/s00411-004-0247-6 PMID: 15316819
27. Heidenreich WF, Tomášek L, Rogel A, Laurier D, Tirmarche M (2004) Studies of radon-exposed miner cohorts using a biologically based model: comparison of current Czech and French data with historic data from China and Colorado. Radiat Environ Biophys 43: 247–56. doi: 10.1007/s00411-004-0266-3 PMID: 15645313
28. van Dillen T, Dekkers F, Bijwaard H, Kreuzer M, Grosche B (2011) Lung cancer from radon: a two-stage model analysis of the WISMUT Cohort, 1955–1998. Radiat Res 175: 119–30. doi: 10.1667/RR2102.1 PMID: 21175354
29. Heidenreich WF, Tomášek L, Grosche B, Leuraud K, Laurier D (2012) Lung cancer mortality in the European uranium miners cohorts analyzed with a biologically based model taking into account radon

measurement error. Radiat Environ Biophys 51: 263–75. doi: 10.1007/s00411-012-0423-z PMID: 22622996

30. Eidemüller M, Jacob P, Lane RSD, Frost SE, Zablotska LB (2012) Lung cancer mortality (1950–1999) among Eldorado uranium workers: a comparison of models of carcinogenesis and empirical excess risk models. PLoS ONE 7: e41431. doi: 10.1371/journal.pone.0041431 PMID: 22936975
31. Jacob V, Jacob P, Meckbach R, Romanov Sa, Vasilenko EK (2005) Lung cancer in Mayak workers: interaction of smoking and plutonium exposure. Radiat Environ Biophys 44: 119–29. doi: 10.1007/s00411-005-0012-5 PMID: 16136318
32. Jacob P, Meckbach R, Sokolnikov M, Khokhryakov VV, Vasilenko E (2007) Lung cancer risk of Mayak workers: modelling of carcinogenesis and bystander effect. Radiat Environ Biophys 46: 383–94. doi: 10.1007/s00411-007-0117-0 PMID: 17562061
33. Brugmans MJ, Bijwaard H, Leenhouts HP (2002) The overrated role of 'promotion' in mechanistic modelling of radiation carcinogenesis. J Radiol Prot 22: A75. doi: 10.1088/0952-4746/22/3A/314 PMID: 12400952
34. Little MP, Haylock RGE, Muirhead CR (2002) Modelling lung tumour risk in radon-exposed uranium miners using generalizations of the two-mutation model of Moolgavkar, Venzon and Knudson. Int J Rad Biol 78: 49–68. doi: 10.1080/09553000110085797 PMID: 11747553
35. Koshurnikova N, Shilnikova N, Okatenko P, Kreslov V, Bolotnikova M, Sokolnikov M, et al. (1999) Characteristics of the cohort of workers at the mayak nuclear complex. Radiat Res 152: 352–363. doi: 10.2307/3580220 PMID: 10477912
36. Anspaugh L, Degteva M, Vasilenko E (2002) Mayak production association: Introduction. Radiat Environ Biophys 41: 19–22. doi: 10.1007/s00411-002-0148-5 PMID: 12014402
37. Gilbert ES, Sokolnikov ME, Preston DL, Schonfeld SJ, Schadilov AE, Vasilenko EK, et al. (2013) Lung cancer risks from plutonium: an updated analysis of data from the Mayak worker cohort. Radiat Res 179: 332–42. doi: 10.1667/RR3054.1 PMID: 23391147
38. Khokhryakov VV, Khokhryakov VF, Suslova KG, Vostrotin VV, Vvedensky VE, Sokolova AB, et al. (2013) Mayak Worker Dosimetry System 2008 (MWDS-2008): assessment of internal dose from measurement results of plutonium activity in urine. Health Phys 104: 366–78. doi: 10.1097/HP.0b013e31827dbf60 PMID: 23439140
39. Vasilenko E, Khokhryakov V, Miller S, Fix J, Eckerman K, Choe D, et al. (2007) Mayak worker dosimetry study: an overview. Health Phys 93: 190. doi: 10.1097/01.HP.0000266071.43137.0e PMID: 17693770
40. Moolgavkar S, Krewski D, Schwarz M (1999) Mechanisms of carcinogenesis and biologically based models for estimation and prediction of risk. IARC Sci Publ: 179.
41. Moolgavkar SH, Dewanji A, Venzon DJ (1988) A stochastic two-stage model for cancer risk assessment. I. The hazard function and the probability of tumor. Risk Anal 8: 383–92. doi: 10.1111/j.1539-6924.1988.tb00502.x PMID: 3201016
42. Heidenreich WF, Luebeck EG, Moolgavkar SH (1997) Some properties of the hazard function of the two-mutation clonal expansion model. Risk Anal 17: 391–9. doi: 10.1111/j.1539-6924.1997.tb00878.x PMID: 9232020
43. Heidenreich W (1996) On the parameters of the clonal expansion model. Radiat Environ Biophys 35: 127–129. doi: 10.1007/BF02434036 PMID: 8792461
44. Eidemüller M, Holmberg E, Jacob P, Lundell M, Karlsson P (2015) Breast cancer risk and possible mechanisms of radiation-induced genomic instability in the Swedish hemangioma cohort after reanalyzed dosimetry. Mutat Res 775: 1–9. doi: 10.1016/j.mrfmmm.2015.03.002 PMID: 25839758
45. Preston DL, Shimizu Y, Pierce Da, Suyama A, Mabuchi K (2003) Studies of mortality of atomic bomb survivors. Report 13: Solid cancer and noncancer disease mortality: 1950–1997. Radiat Res 160: 381–407. doi: 10.1667/RR3049 PMID: 12968934
46. McCullagh P, Nelder JA (1991) Generalized linear models. Chapman & Hall, London, 2nd edition.
47. James F, Roos M (1975) Minuit—a system for function minimization and analysis of the parameter errors and correlations. Cancer Causes Control 10: 343–367.
48. Akaike H (1974) A new look at the statistical model identification. IEEE Trans Automat Contr 19: 716–723. doi: 10.1109/TAC.1974.1100705
49. Heidenreich WF, Brugmans MJP, Little MP, Leenhouts HP, Paretzke HG, Morin M, et al. (2000) Analysis of lung tumour risk in radon-exposed rats: an intercomparison of multi-step modelling. Radiat Environ Biophys 39: 253–264. doi: 10.1007/s004110000075 PMID: 11200969
50. Kopp-Schneider A, Haertel T, Burkholder I, Bannasch P, Wesch H, Groos J, et al. (2006) Investigating the formation and growth of alpha-particle radiation-induced foci of altered hepatocytes: a model-based approach. Radiat Res 166: 422–30. doi: 10.1667/RR3526.1 PMID: 16881743

51. Chao DL, Eck JT, Brash DE, Maley CC, Luebeck EG (2008) Preneoplastic lesion growth driven by the death of adjacent normal stem cells. PNAS 105: 15034–15039. doi: 10.1073/pnas.0802211105 PMID: 18815380
52. Heidenreich WF, Atkinson M, Paretzke HG (2001) Radiation-induced cell inactivation can increase the cancer risk. Radiat Res 155: 870–872. doi: 10.1667/0033-7587(2001)155%5B0870:RICICI%5D2.0.CO;2 PMID: 11352771
53. Bijwaard H, Brugmans M, Schöllnberger H (2006) Can promotion of initiated cells be explained by excess replacement of radiation-inactivated neighbor cells? Radiat Res 744: 741–744. doi: 10.1667/RR3548.1
54. Heidenreich WF, Paretzke HG (2008) Promotion of initiated cells by radiation-induced cell inactivation. Radiat Res 170: 613–7. doi: 10.1667/RR0957.1 PMID: 18959457
55. Madas BG, Varga K (2014) Biophysical modelling of the effects of inhaled radon progeny on the bronchial epithelium for the estimation of the relationships applied in the two-stage clonal expansion model of carcinogenesis. Radiat Prot Dosim 159: 237–41. doi: 10.1093/rpd/ncu125
56. National Research Council (1999) Health Effects of Exposure to Radon: BEIR VI. (Natl. Acad. Press, Washington, DC).
57. Harley NH, Chittaporn P, Meyers OA, Robbins ES (1996) A biological model for lung cancer risk from 222-Rn exposure. Environ Int 22: 977–984. doi: 10.1016/S0160-4120(96)00210-3
58. Balásházy I, Farkas A, Madas BG, Hofmann W (2009) Non-linear relationship of cell hit and transformation probabilities in a low dose of inhaled radon progenies. J Radiol Prot 29: 147–62. doi: 10.1088/0952-4746/29/2/003 PMID: 19454792
59. Curtis S, Hazelton W, Luebeck E, Moolgavkar S (2004) From mechanisms to risk estimation—bridging the chasm. Adv Space Res 34: 1404–1409. doi: 10.1016/j.asr.2004.03.011 PMID: 15881782
60. Trosko JE, Chang CC, Upham BL, Tai MH (2005) Low-dose ionizing radiation: induction of differential intracellular signalling possibly affecting intercellular communication. Radiat Environ Biophys 44: 3–9. doi: 10.1007/s00411-005-0269-8 PMID: 15821925
61. Kundrát P, Friedland W (2014) Impact of intercellular induction of apoptosis on low-dose radiation carcinogenesis. submitted to Radiat Prot Dos.
62. Shuryak I, Brenner DJ, Ullrich RL (2011) Radiation-induced carcinogenesis: mechanistically based differences between gamma-rays and neutrons, and interactions with DMBA. PLoS ONE 6: e28559. doi: 10.1371/journal.pone.0028559 PMID: 22194850
63. Brenner DJ, Little JB, Sachs RK (2001) The Bystander Effect in Radiation Oncogenesis: II. A Quantitative Model. Radiat Res 155: 402–408. doi: 10.1667/0033-7587(2001)155%5B0402:TBEIRO%5D2.0.CO;2
64. Sawant SG, Randers-Pehrson G, Geard CR, Brenner DJ, Hall EJ (2001) The bystander effect in radiation oncogenesis: I. transformation in C3H 10T 1/2 cells in vitro can be initiated in the unirradiated neighbors of irradiated cells. Radiat Res 155: 397–401.
65. Zhou H, Suzuki M, Randers-Pehrson G, Vannais D, Chen G, Trosko JE, et al. (2001) Radiation risk to low fluences of alpha particles may be greater than we thought. PNAS 98: 14410–5. doi: 10.1073/pnas.251524798 PMID: 11734643
66. Abdelrazzak AB, Stevens DL, Bauer G, O'Neill P, Hill MA (2011) The role of radiation quality in the stimulation of intercellular induction of apoptosis in transformed cells at very low doses. Radiat Res 176: 346–355. doi: 10.1667/RR2509.1 PMID: 21663396
